# The Impact of Inadequate Terminal Disinfection on an Outbreak of Imipenem-Resistant *Acinetobacter baumannii* in an Intensive Care Unit

**DOI:** 10.1371/journal.pone.0107975

**Published:** 2014-09-25

**Authors:** Wei-Lun Liu, Hsueh-Wen Liang, Mei-Feng Lee, Hsin-Lan Lin, Yu-Hsiu Lin, Chi-Chung Chen, Ping-Chin Chang, Chih-Cheng Lai, Yin-Ching Chuang, Hung-Jen Tang

**Affiliations:** 1 Department of Intensive Care Medicine, Chi Mei Medical Center, Liouying, Tainan, Taiwan; 2 Department of Nursing, Internal Medicine, and Intensive Care Medicine, Chi Mei Medical Center, Liouying, Tainan, Taiwan; 3 Department of Medical Research, Chi Mei Medical Center, Tainan, Taiwan; 4 The Committee of Infection Control, Chi Mei Medical Center, Liouying, Tainan, Taiwan; 5 Institute of Biotechnology, National Cheng Kung University, Tainan, Taiwan; 6 Department of Internal Medicine, Chi Mei Medical Center, Liouying, Tainan, Taiwan; 7 Department of Medicine, Chi Mei Medical Center, Tainan, Taiwan; 8 Department of Health and Nutrition, Chia Nan University of Pharmacy and Science, Tainan, Taiwan; Queen Mary Hospital, the University of Hong Kong, Hong Kong

## Abstract

**Background:**

This study was conducted to investigate an outbreak caused by imipenem-resistant *Acinetobacter baumannii* (IRAB) in a medical intensive care unit (ICU) in a regional hospital.

**Methods:**

In response to an IRAB outbreak from October 2012 to February 2013, we developed several infection control measures, including an extensive review process of environmental cleaning and disinfection, and used molecular methods to identify each clinical and environmental IRAB isolate.

**Results:**

During this five-month period, 22 patients were colonized with IRAB and 18 patients had IRAB infections. The in-hospital mortality rate was significantly higher among patients with infections rather than colonizations (44.4% vs 9.1%, p = 0.028). Additionally, nine environmental specimens, including five specimens collected after terminal disinfection, were positive for IRAB. 12 environmental isolates and 28 of 36 available clinical isolates belonged to one unique pulsotype A, which was confirmed by molecular methods. We found the concentration of disinfectant, 0.08% sodium hypochlorite, was inadequate. After correcting the environmental cleansing methods, the surveillance study showed no further IRAB isolates on the control panel surfaces of the medical equipment or in patients in the ICU. Additionally, an *in* *vitro* study of IRAB immersed in different concentrations of sodium hypochlorite showed that 0.5% sodium hypochlorite eradicates IRAB after 30 seconds of inoculation, but 0.08% sodium hypochlorite only reduces the bacterial load.

**Conclusions:**

This study highlights the importance of the preparation of disinfectants to adequately achieve environmental disinfection in the control of IRAB outbreaks in the ICU.

## Introduction

*Acinetobacter baumannii*, a non-fermenting Gram-negative coccobacillus has become an important nosocomial pathogen, especially in intensive care units (ICUs). Moreover, the increasing appearance of multiple drug resistance in this pathogen, especially carbapenem-resistance, limits the therapeutic antibiotic options for patients infected with *A. baumannii*. Most importantly, this multi-drug resistant (MDR) pathogen can cause healthcare-associated infections and can increase mortality and the length of stay in the ICU [1], [2].

Because *A. baumannii* has a great ability to colonize humans and environmental surfaces [3], [4], it is difficult to eradicate this pathogen from the environment. In addition to colonization, it can cause life-threatening human infections, especially in immunocompromised and critically ill patients. Therefore, MDR *A. baumannii* (MDRAB) remains a global issue in public health despite aggressive infection control measures to avoid nosocomial acquisition and further dissemination.

Recently, we noted an outbreak of imipenem-resistant *A. baumannii* (IRAB) in an ICU at a regional hospital in southern Taiwan. To prevent further outbreaks and their accompanying risks, we extensively reviewed our infection control policy and designed a care bundle for restricting the colonization and spread of IRAB.

## Materials and Methods

### Setting

Chi Mei Medical Center, Liouying campus is a 900-bed regional hospital located in southern Taiwan with a 16-bed medical ICU. In October 2012, a computer-based infection control system for the analysis of microbiologic and clinical data detected an outbreak of IRAB. All of the case with IRAB was identified by microbiology department initially and the information was transmitted to infection control nurses. After checking electrical chart for collecting the clinical information, the outbreak of IRAB was confirmed by the committee of infection control. Case definitions for infection or colonizations followed the guidelines published by the Centers for Disease Control and Prevention [5]. To investigate this outbreak, we conducted active surveillance and molecular characterization of IRAB isolates from the environment and patients who were either colonized or infected. An ethics approval was obtained from Institutional Review Board of Chi Mei Medical Center after the investigation of the outbreak.

### Microbiological investigation

*A. baumannii* isolates were identified by conventional biochemical tests and by two commercial identification kits, Api20NE (bioMerieux, Marcy I’Etoile, France) and the Phoenix System (Becton Dickson, Sparks, MD). Isolates were classified as susceptible or resistant (including an intermediate category) by broth microdilution methods according to Clinical and Laboratory Standards Institute (CLSI) guidelines [6]. IRAB was defined as *A. baumannii* isolates resistant to imipenem. Environmental specimens (including bedrails, monitors, respirators, bedside desks, and bedside sinks) were collected on moistened gauze, incubated overnight in tryptic soy broth and then subcultured onto blood agar and MacConkey agar. The hands of personnel caring for these patients were randomly sampled during work and before hand washing with a cotton swab moistened with brain heart infusion (BHI) broth [7]. To avoid the concern that the staffs will clean their hands vigorously before the sampling, infection control nurses were responsible for surveillance culture and they must perform the sampling culture without informing the staff.

### Infection control intervention

After the detection of the outbreak, a team including intensivists, ICU nurses, microbiologists, house-keeping staff and infectious control specialists was organized. The team developed a plan regarding the control of the outbreak based on the consensus of infection control team. We developed several infection control measures, including (1) enhancing contact isolation of all IRAB patients and empirical contact isolation of patients at high risk for acquiring IRAB (including hospitalization in the preceding 90 days, residency in a nursing home, extended-care facility, and receiving broad-spectrum antimicrobial therapy in the preceding 90 days); (2) enhancing contact precautions to interrupt transmission, including hand washing and the use of disposable gloves and gowns; (3) monitoring isolation and hand hygiene adherences; (4) reviewing the process of environmental cleaning and disinfection; (5) instituting an educational program for healthcare workers; (6) establishing periodic environmental cultures to identify contaminated surfaces that might constitute a source of indirect *A. baumannii* spread; (7) using molecular methods to identify available IRAB clinical and environmental isolates; and (8) discontinue isolation when there were three successive negative cultures from the original site of clinical specimen positive culture for IRAB such as respiratory specimen, urine, or wounds, if available during the follow-up period.

### Pulsed-field gel electrophoresis analysis

The genetic relatedness studies of IRAB isolates were performed by pulsed-field gel electrophoresis (PFGE) using ApaI (New England Biolabs, Ipswich, MA, USA) as previously described with slight modifications. [8] Briefly, the bacteria were grown at 37°C overnight on Trypticase soy agar with 2% sheep blood (BBL) and suspended in 2 mL of cell suspension buffer (100 mM Tris, 100 mM EDTA, pH 8.0) to a concentration of 10^9^ CFU/mL. Then, the bacterial suspension was mixed with an equal volume of 1% Pulsed Field Certified Agarose (Bio-Rad Laboratories, Hercules, CA, USA) and allowed to solidify in a 100-µL plug mold (Bio-Rad Laboratories, Hercules, CA, USA). The gel plugs were then lysed, washed, and digested with the restriction enzyme ApaI. PFGE was performed with a CHEF Mapper system (Bio-Rad Laboratories, Hercules, CA, USA) at 14°C and a field strength of 6 V/cm with a pulse time of 1–25 seconds for 22 hours. A bacteriophage lambda ladder PFGE marker (New England Biolabs, Ipswich, MA, USA) was used as a reference marker. Cluster analyses were performed using BioNumerics software, version 3.5 (Applied Maths, Sint-Martens-Latem, Belgium). DNA relatedness was calculated using the Dice coefficient with a tolerance of 1%. PFGE profiles were interpreted as epidemiological relatedness according to the criteria suggested by Tenover et al. [9] Strains with more than 85% similarity values of PFGE profiles were considered as closely related strains in this study.

### *In vitro* study of the effect of sodium hypochlorite on IRAB isolates

In this *in* *vitro* study, 6 isolates were randomly select from different pulsed-field gel electrophoresis (PFGE) samples. After overnight culture in tryptic soy broth (TSB) (Difco Co; Becton Dickinson, Sparks, MD), the concentration was adjusted using the 0.5 Mc Farland standard (1×10^8^ CFU/mL). The culture was pelleted in 1 mL bacterial suspension by centrifugation, and the supernatant was replaced with normal saline or 0.5%, 0.2%, 0.1%, 0.08% or 0.05% sodium hypochlorite. The cultures were then incubated for 30 and 60 seconds at 20°C. After each period of time, 9 mL of neutralizing broth (D/E Neutralizing Broth, Difco Co) was added to terminate the antimicrobial action of the test agents. Ten-fold serial dilutions were performed in reduced transport fluid. Each dilution was plated onto TSA plates. The plates were then incubated for 24 hours at 37°C, and bacterial numbers were calculated. Controls were exposed to sterile saline for the same periods. Three replicates were performed for each antimicrobial agent and control.

### Drug Definition

Extended-spectrum cephalosporins included ceftriaxone, flomoxef, ceftazidime, and cefpirome. Extended-spectrum β-lactam-β-lactamase inhibitor combinations included piperacillin and piperacillin-tazobactam. Carbapenems included imipenem, meropenem, and ertapenem, and fluoroquinolones included ciprofloxacin, moxifloxacin and levofloxacin.

### Statistical analysis

Continuous variables are expressed as the mean±standard deviation. Continuous variables were compared using the Wilcoxon rank-sum test or Student’s independent *t* test, as appropriate. Categorical variables were compared using the chi-square test or Fisher’s exact test. All statistical analyses were conducted using the statistical package SPSS for Windows (Version 11.0, SPSS, Chicago, Il, USA).

## Results

### Clinical characteristics of patients with IRAB colonization and infection

**Figure 1:**
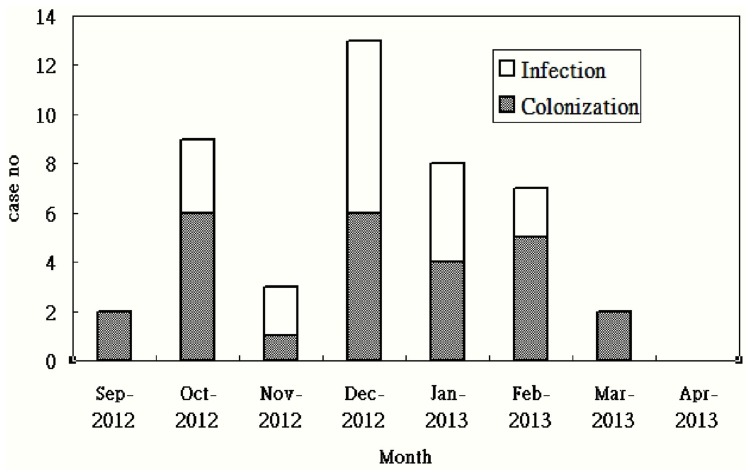
Case numbers of imipenem-resistant *Acinetobacter baumannii* colonization and infections over time.

During a five-month period, a total of 40 patients yielded clinical specimens that were positive for IRAB isolates. The clinical characteristics of these 40 patients are summarized in table 1. The mean age was 69.2 years, and 62.5% (n = 25) of patients were ≥65 years. Diabetes mellitus was the most common comorbidity, followed by malignancy. An endotracheal aspirate was the most common clinical specimen positive for IRAB, followed by the tip of a central venous catheter, blood, and catheterization urine. Except for one patient, most of the patients (n = 39) had received broad-spectrum antibiotics (such as extended-spectrum cephalosporins, extended-spectrum β-lactam-β-lactamase inhibitor combinations, carbapenems, and fluoroquinolones), especially fluoroquinolones. A total of 22 patients (55%) were defined as being colonized by IRAB, and the other 18 patients were defined as having IRAB infections (Figure 1). Overall in-hospital mortality was 25%.

**Table 1:** Demographic characteristics of patients with positive isolates for imipenem-resistant *Acinetobacter baumannii* (IRAB).

Variable	No of patients (%) n = 40
Age (years), mean ± SD	69.2±16.3
Male (%)	24 (60.0)
Underlying disease	
Diabetes mellitus	14 (35.0)
Cancer	13 (32.5)
Stroke	7 (17.5)
Chronic kidney disease	5 (12.5)
Liver cirrhosis	4 (10.0)
Connective tissue disease	2 (5.0)
Use of steroid	6 (15.0)
Use of immunosuppressant	4 (10.0)
Site of isolates	
Endotracheal aspirate	30 (75.0)
Catheter tip	4 (10.0)
Blood	3 (15.0)
Urine	2 (5.0)
Wound	2 (5.0)
Device	
Endotracheal tube	37 (92.5)
Central venous catheter	32 (80.0)
Port-A catheter	7 (17.5)
Double lumen catheter	4 (10.0)
AV shunt	1 (2.5)
Abdominal drainage	4 (10.0)
Pleural drainage	2 (5.0)
Total parenteral nutrition	3 (7.5)
Previous use of antibiotic in the preceding 90 days	
Fluoroquinolones	27 (67.5)
Carbapenem	17 (42.5)
Extended-spectrum cephalosporin	16 (40.0)
Extended-spectrum β-lactam-β-lactamase inhibitor combinations	7 (17.5)
Hospital stay before acquisition of IRAB (days)	14.4±9.4
ICU stay before acquisition of IRAB (days)	10.1±5.5
Clinical significance	
Colonization	22 (55.0)
Ventilator associated pneumonia	15 (30.0)
Central venous catheter related infection	2 (10.0)
Skin and soft tissue infection	1 (5.0)
Outcome	
In-hospital mortality	10 (25.0)

### Comparison between patients with IRAB colonization and infections

Among the 18 patients who had IRAB infections, ventilator-associated pneumonia was the most common type of infection. The comparison between patients with IRAB colonizations and infections is summarized in table 2. Although patients with infections were older than patients with colonizations, this difference was not significant (mean age in year, 74.3 vs 65.0, p = 0.0714). However, we noted that the patients with colonizations were more likely to have received 3^rd^ or 4^th^ generation cephalosporins than patients with infections (59.1% vs 16.7%, p = 0.0165). In contrast, the patients with infections had longer ICU stays before the acquisition of IRAB than patients who were colonized (mean duration in days, 11.2 vs 9.2, p = 0.0498). Importantly, the patients with infections had a higher in-hospital mortality rate than patients who were colonized (44.4% vs 9.1%, p = 0.0279).

**Table 2:** Comparison between patients with imipenem-resistant *Acinetobacter baumannii* (IRAB) colonization and infections.

Variable	No of patients with colonization (n = 22)	No of patients with infections (n = 18)	*P* value
Age (years), mean ± SD	65.0±17.4	74.3±13.5	0.0714
Male (%)	11 (50.0)	13 (72.2)	0.2707
Underlying disease			
Diabetes mellitus	8 (36.4)	6 (33.3)	0.8976
Cancer	8 (36.4)	5 (27.8)	0.8116
Stroke	3 (13.6)	4 (22.2)	0.7687
Chronic kidney disease	3 (13.6)	2 (11.1)	0.8081
Liver cirrhosis	2 (9.1)	2 (11.1)	0.7490
Connective tissue disease	2 (9.1)	0 (0.0)	0.5590
Use of steroid	3 (13.6)	3 (16.7)	0.8635
Use of immunosuppressant	3 (13.6)	1 (5.6)	0.7571
Device			
Endotracheal tube	19 (86.4)	18 (100.0)	0.3065
Central venous catheter	15 (68.2)	17 (94.4)	0.0962
Port-A catheter	4 (18.2)	3 (16.7)	0.7689
Double lumen catheter	2 (9.1)	2 (11.1)	0.7490
AV shunt	1 (4.5)	0 (0.0)	0.9112
Abdominal drainage	2 (9.1)	2 (11.1)	0.7490
Pleural drainage	1 (4.5)	1 (5.6)	0.5683
Total parenteral nutrition	1 (4.5)	2 (11.1)	0.8529
Previous use of antibiotic			
Fluoroquinolones	15 (68.2)	12 (66.7)	0.8114
Carbapenem	11 (50.0)	6 (33.3)	0.4584
Extended-spectrum cephalosporin	13 (59.1)	3 (16.7)	**0.0165**
Extended-spectrumβ-lactam-β-lactamase inhibitor combinations	5 (13.6)	2 (22.2)	0.7687
Hospital stay before acquisition of IRAB (days)	14.7±11.1	13.9±7.2	0.7936
ICU stay before acquisition of IRAB (days)	9.2±4.8	11.2±5.5	**0.0498**
Outcome			
In-hospital mortality	2 (9.1)	8 (44.4)	**0.0279**

### Microbiologic investigations

**Figure 2:**
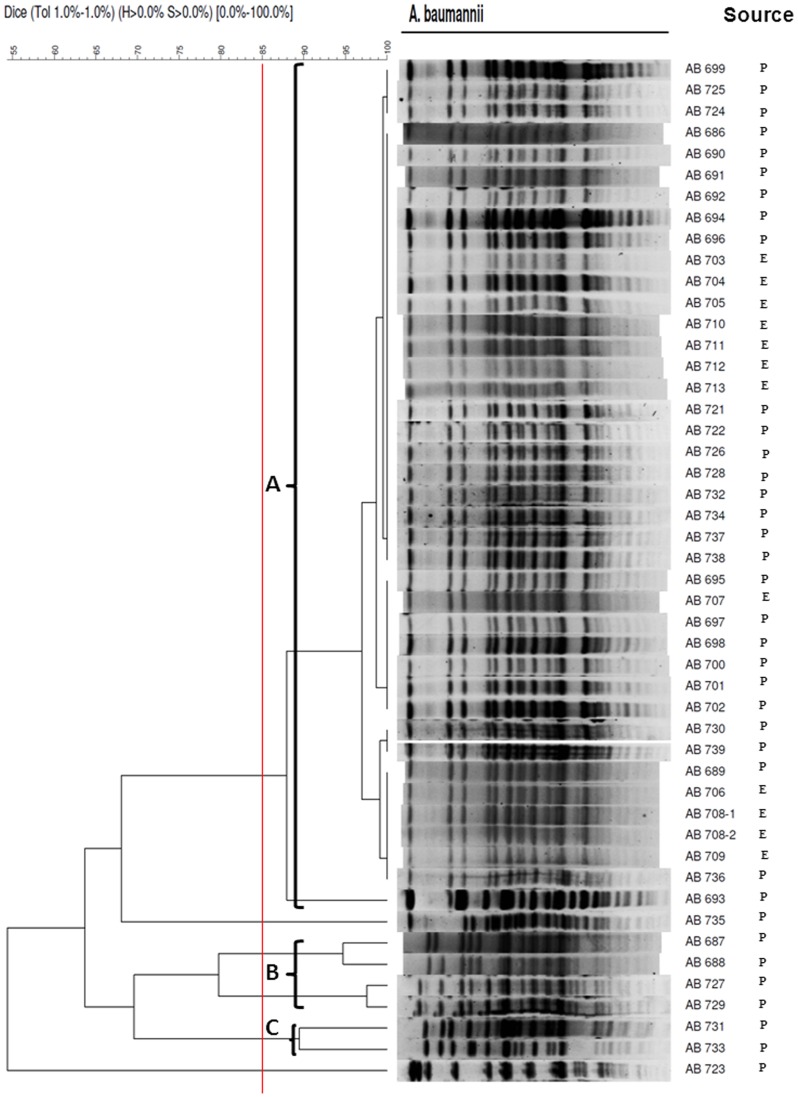
Pulsed-field gel electrophoresis (PFGE) profiles of 48 imipenem-resistant *A. baumannii* isolates digested with ApaI. Red line indicated the 85% similarity values of PFGE profiles. P: clinical isolate from patient; E: environmental isolate.

Of 22 environmental samplings during the outbreak investigation, a total of twelve environmental isolates which including five specimens collected after terminal disinfection were positive for IRAB. All environmental isolates and the 36 clinical isolates that were available from patients were sent for molecular study. We found that all of the environmental isolates and most of the clinical isolates (n = 28) belonged to one unique pulsotype A. Additionally, four isolates were belonged to pulsotype B, and two clinical isolates were belonged to pulsotype C (Figure 2).

### Infection control process

After the IRAB outbreak was identified, we performed environmental surveillance cultures. We found IRAB in isolates grown from sampling the bedside desks and the surface of a body weight scale, even after cleaning and terminal disinfection. This compelled us to extensively review the entire process of terminal disinfection. It appears that for some reason, a too low concentration was used. Therefore, we corrected the procedure for preparing disinfectant solutions and reinforced the need for adherence to disinfection protocols. After the surveillance study and the reinforcement of the correct process for environmental cleansing, further testing of 40 environmental specimens showed no IRAB isolates from samples on control panel surfaces of the medical equipment and of the patients in the ICU. Additionally, the hand hygiene compliance of the ICU staff increased from 48% in the pre-intervention period to 89% in the post-intervention period.

### *In vitro* study of the effect of sodium hypochlorite on IRAB isolates

**Figure 3:**
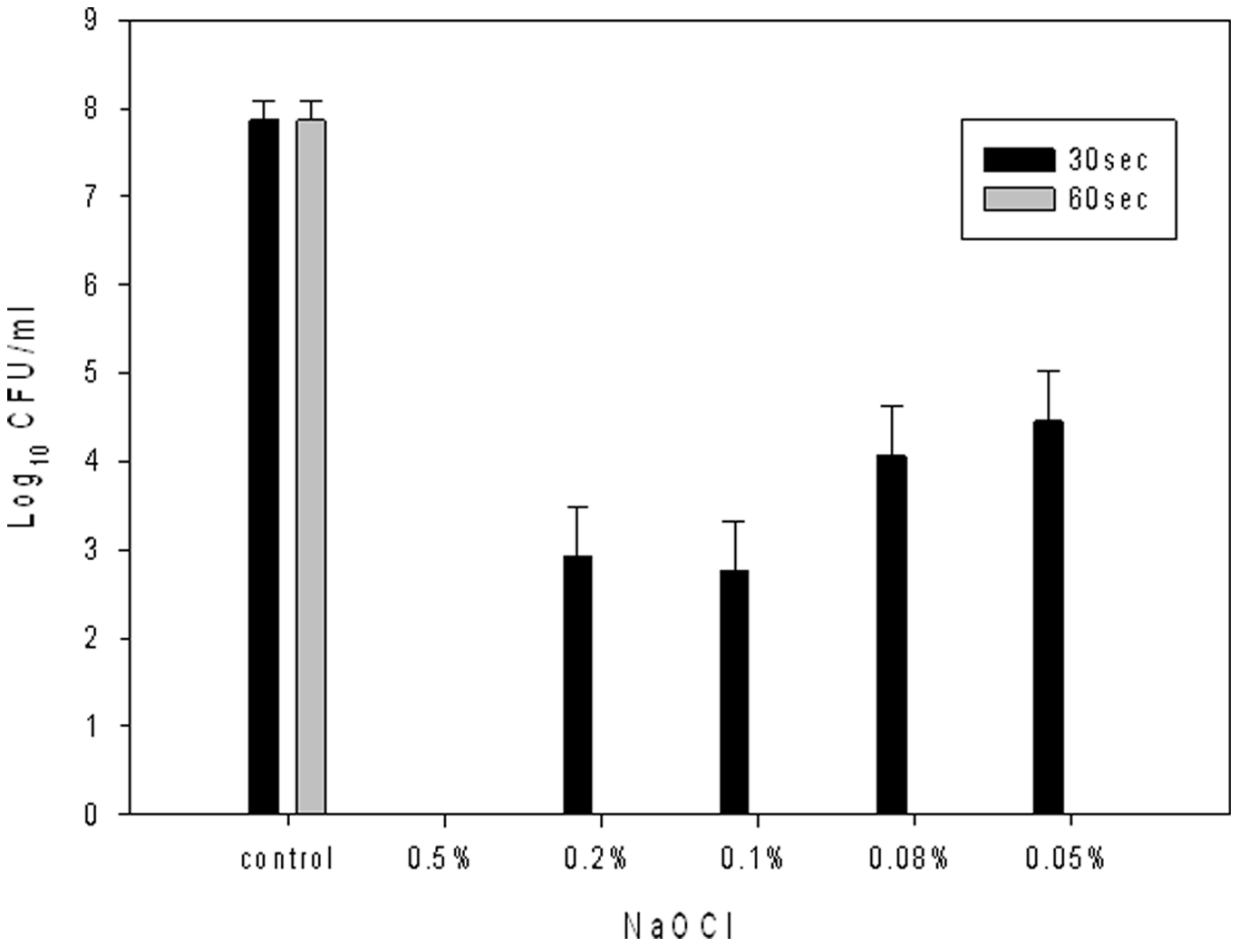
The number of imipenem-resistant *Acinetobacter baumannii* in different concentration of sodium hypochlorite after 30 and 60 seconds.

After 30 seconds of treatment, only 0.5% sodium hypochlorite eradicated all six isolates. 0.2%∼0.05% sodium hypochlorite can only reduce the bacterial number to 10^3^∼10^5^ CFU/ml. The bacteria number after 60 seconds in the various solutions of sodium hypochlorite was below detectable levels (Figure 3).

## Discussion

Several significant findings arose from our investigation of an outbreak of IRAB in an ICU. First, we showed the presence of a large outbreak of IRAB that lasted for five months using a molecular approach. The study showed that this outbreak was strongly associated with the use of incorrect procedures during terminal disinfections. The wrong protocol for preparing the disinfectant, sodium hypochlorite, meant that the concentration of disinfectant (0.08%) was inadequate as a bactericidal agent. Several specimens from the environmental surveillance grew IRAB even after terminal disinfection. After extensively reviewing every step of the infection control protocol and correcting the process for terminal disinfection, no new IRAB cases were identified. In addition, an *in* *vitro* study of IRAB using different concentrations of sodium hypochlorite confirmed that only a 0.5% sodium hypochlorite solution eradicates IRAB after 30 seconds of exposure. Concentrations less than 0.5% cannot effectively kill all isolates unless the clinical isolates are exposed to the cleaning agent for more than 60 seconds. Therefore, although the outbreak of IRAB, causing 12 and 28 episodes of infection and colonization in the study unit may be multifactorial, both of the clinical and laboratory findings suggest that inadequate terminal disinfection should be one of most important factors. Such a costly mistake reminds us of the importance of infection control measures [10], including the preparation of disinfectant. As our experience is limited, we cannot recommend the appropriate concentration of sodium hypochlorite solution for environmental cleaning all of the important nosocomial pathogens. However, our finding may indicate that only sodium hypochlorite solution with the concentration of at least 0.5% can be useful for terminal disinfection for IRAB.

At the beginning of the intervention, the hand hygiene compliance of the ICU staff was less than 50%. It is difficult to totally exclude the possibility that IRAB was spread patient-to-patient by contaminated hands; however, we were unable to isolate IRAB on the hands of the ICU staff during the environmental surveillance despite we only sampled a very small portion of the actual patient contacts. Therefore, the impact of poor hand hygiene on this outbreak may be limited. In addition, hand hygiene compliance rapidly improved after education and monitoring of hand hygiene. By the end of the outbreak, the compliance was nearly 90%. This improvement in hand hygiene may be due to repeated education programs and the monitoring of the contact precautions.

In this study, the patients with IRAB had varying risk factors, including immunocompromised comorbidities, antecedent broad-spectrum antimicrobial therapy and recent invasive procedures. This finding is consistent with previous studies showing that the risk factors for the acquisition of multi-drug resistant *A. baumannii* include malignancy, recent exposure to antibiotics, mechanical ventilation, and higher disease severity measured by the Acute Physiology and Chronic Health Evaluation (APACHE) score [11]–[19]. This suggests that while caring for patients at high risk, healthcare workers should strictly follow the standard care procedures to prevent patients from acquiring multi-drug resistant organisms.

In addition, we were unable to find any significant differences in the underlying conditions, including age, gender, and comorbidities among patients with IRAB infections versus colonizations in the present study. However, we noted that patients with infections had longer ICU stays before the acquisition of IRAB than patients who were colonized. Furthermore, as previous reported in Spain [20], patients with IRAB infections had significantly higher mortality rates than patients who were colonized (44.4% vs 9.1%). Moreover, our findings and other recent studies suggest that multi-drug resistant *A. baumannii* infections are associated with high mortality [19]–[22].

In conclusion, this study highlights the importance of adequate environmental disinfection and the correct preparation of disinfectants in the control of IRAB outbreaks in ICUs. After eradicating environmental contamination through effective terminal disinfection, outbreaks of IRAB can be controlled, as we demonstrated. If outbreaks of IRAB are not well controlled, they are associated with high mortality.
